# Short-term synaptic depression can increase the rate of information transfer at a release site

**DOI:** 10.1371/journal.pcbi.1006666

**Published:** 2019-01-02

**Authors:** Mehrdad Salmasi, Alex Loebel, Stefan Glasauer, Martin Stemmler

**Affiliations:** 1 Graduate School of Systemic Neurosciences, Ludwig-Maximilians-Universität, Munich, Germany; 2 Bernstein Center for Computational Neuroscience, Munich, Germany; 3 German Center for Vertigo and Balance Disorders, Ludwig-Maximilians-Universität, Munich, Germany; 4 Department of Biology II, Ludwig-Maximilians-Universität, Munich, Germany; 5 Chair of Computational Neuroscience, Brandenburg University of Technology Cottbus-Senftenberg, Senftenberg, Germany; University of Edinburgh, UK, UNITED KINGDOM

## Abstract

The release of neurotransmitters from synapses obeys complex and stochastic dynamics. Depending on the recent history of synaptic activation, many synapses depress the probability of releasing more neurotransmitter, which is known as synaptic depression. Our understanding of how synaptic depression affects the information efficacy, however, is limited. Here we propose a mathematically tractable model of both synchronous spike-evoked release and asynchronous release that permits us to quantify the information conveyed by a synapse. The model transits between discrete states of a communication channel, with the present state depending on many past time steps, emulating the gradual depression and exponential recovery of the synapse. Asynchronous and spontaneous releases play a critical role in shaping the information efficacy of the synapse. We prove that depression can enhance both the information rate and the information rate per unit energy expended, provided that synchronous spike-evoked release depresses less (or recovers faster) than asynchronous release. Furthermore, we explore the theoretical implications of short-term synaptic depression adapting on longer time scales, as part of the phenomenon of metaplasticity. In particular, we show that a synapse can adjust its energy expenditure by changing the dynamics of short-term synaptic depression without affecting the net information conveyed by each successful release. Moreover, the optimal input spike rate is independent of the amplitude or time constant of synaptic depression. We analyze the information efficacy of three types of synapses for which the short-term dynamics of both synchronous and asynchronous release have been experimentally measured. In hippocampal autaptic synapses, the persistence of asynchronous release during depression cannot compensate for the reduction of synchronous release, so that the rate of information transmission declines with synaptic depression. In the calyx of Held, the information rate per release remains constant despite large variations in the measured asynchronous release rate. Lastly, we show that dopamine, by controlling asynchronous release in corticostriatal synapses, increases the synaptic information efficacy in nucleus accumbens.

## Author summary

Fatigue is an intrinsic property of living systems and synapses are no exception. Synaptic depression reduces the ability of synapses to release vesicles in response to an incoming action potential. Whether synaptic depression simply reflects the exhaustion of neuronal resources or whether it serves some additional function is still an open question. We ask how synaptic depression modulates the information transfer between neurons by keeping the synapse in an appropriate operating range. Using a tractable mathematical model for synaptic depression of both synchronous spike-evoked and asynchronous release of neurotransmitter, we derive a closed-form expression for the mutual information rate. Depression, it turns out, can both enhance or impair information transfer, depending on the relative level of depression for synchronous spike-evoked and asynchronous releases. We also study the compromise a synapse makes between its energy consumption and the rate of information transmission. Interestingly, we show that synaptic depression can regulate energy use without affecting the information (measured in bits) per synaptic release. By applying our mathematical framework to experimentally measured synapses, we show that some synapses can compensate for intrinsic variability in asynchronous release rates; moreover, we show how neuromodulators such as dopamine act to improve the information transmission rate.

## Introduction

Chemical synapses are the main conduits of information in the nervous system [1]. At such synapses, a presynaptic action potential induces docked vesicles, packed with neurotransmitters, to release with a certain probability. A vesicle release leads to a local postsynaptic dendritic voltage fluctuation, which, in turn, can lead to the generation or inhibition of a postsynaptic action potential, depending on whether the synapse is excitatory or inhibitory [2]. Due to the stochastic nature of vesicle release, a release failure may occur upon the arrival of an action potential; alternatively, a synapse can release a vesicle asynchronously [3], or a spontaneous release may occur even without an action-potential [4]. In addition, at many synaptic connections, the release probability is not constant, but exhibits short-term dynamics on time scales of tens to hundreds of milliseconds [5–8]. The prevalent dynamics consists of short-term depression, in which the release probability instantaneously decrease upon vesicle release, and gradually recovers back during quiescent periods [9, 10]. Several hypotheses have been suggested for the functional role of short-term depression, such as temporal filtering of presynaptic spike trains [11, 12], decorrelation and compression of inputs [13], adaptation to identical stimuli [14], and regulation of information transfer [15–17].

In particular, the rate of information transfer at a synapse is an essential measure of its efficacy. Synaptic information efficacy has been studied numerically [16, 18, 19], its capacity bounded analytically [20], and, in combination with numerical methods, some approximations of the information rate have been derived [21, 22]. However, the complexity and dynamics of synaptic transmission have forced the use of elaborate models for information transmission and have proved to be an obstacle to the derivation of a closed form expression for synaptic information efficacy. Furthermore, the energy-efficiency of information transfer at synapses has yet to be studied analytically. Stronger depression and slower recovery reduce both the use of metabolic energy and the release probability, so the parameters of depression tune the information-energy trade-off in neurons [23]. Moreover, it remains elusive how the stochastic properties of the synapse, in particular asynchronous and spontaneous release, modulate the energy-information regime of the synapse.

To address these issues, we present a tractable, mathematical multi-state model for short-term depression at a single release site. The stochastic relation between spikes and synaptic releases is represented by a binary asymmetric channel for each state. The model allows us to distinguish between the synaptic release mechanisms, namely synchronous spike-evoked release and asynchronous (including spontaneous) release; and the current state (release probability) of the channel is determined by the release history. Building upon an earlier model [24], the introduction of multiple states allows the present model to capture the gradual recovery of the site after a release, and thus connects to classic models of depression based on differential equations [22].

Using this model, we derive analytical closed-from expression for the mutual information rate of the release site under depression. We also consider the energy consumption of the synapse and calculate the energy-normalized information rate of the release site. We study the impact of depression parameters on the information rate and information-energy compromise of the synapse. Our findings clarify how the level of depression and the recovery time constant modulate the information rate of the release site. We subsequently assess the impact of asynchronous and spontaneous release on the information rate of a synapse during short-term depression. The joint analysis of short-term depression and asynchronous release reveals the modulatory impact of stochastic features of the synapse on the functional role of depression. Our results present a new categorization for synapses which is based on the increase/decrease of information rate and energy-normalized information rate of the synapse during short-term depression.

We apply our framework to the experimental measurements and evaluate the information efficacy of three types of synapses: hippocampal autaptic synapses, calyx of Held, and corticostriatal synapses in nucleus accumbens. Our analysis leads to compelling results about the role of asynchronous release and modulatory neurotransmitters (like dopamine) in changing synaptic information efficacy.

## Methods

**Fig 1:**
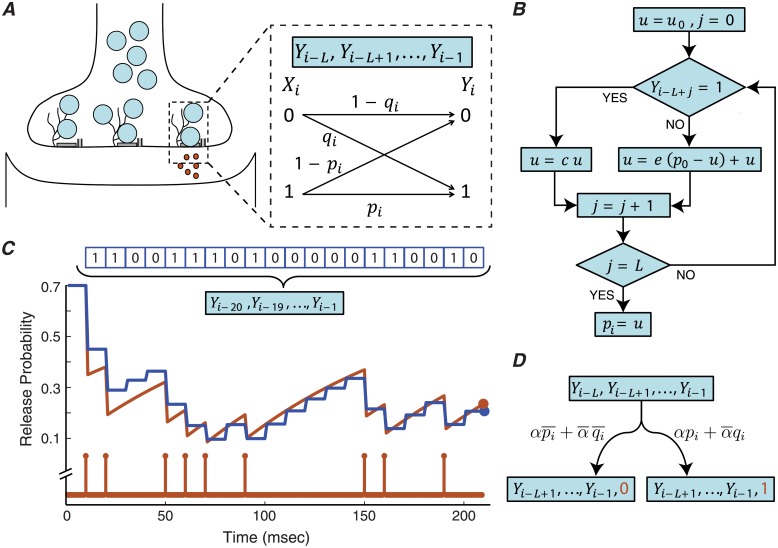
(A) A binary asymmetric channel with a finite memory is used to model the release site under short-term depression. The synchronous release probability, *p*_*i*_, and the asynchronous release probability, *q*_*i*_, are determined based on the previous *L* release outcomes of the release site. (B) The algorithm for calculating the synchronous spike-evoked release probability, *p*_*i*_, given the last *L* release outcomes, (*Y*_*i*−*L*_, *Y*_*i*−*L*+1_, …, *Y*_*i*−1_). The asynchronous release probability, *q*_*i*_, can be derived similarly by substituting *c*, *e* and *p* with *d*, *f* and *q* respectively. We assume that the model parameters (*α*, *c*, *d*, *e*, *f*, *p*_0_, *q*_0_) are strictly greater than zero and less than one. The seed value of the algorithm, *u*_0_, is set to *p*_0_ for synchronous release and *q*_0_ for asynchronous release. (C) The performance of the algorithm is compared with the stochastic model of depression [22]. The stochastic model is based on the differential equation $\frac{dp_{r}}{dt}=\frac{p_{0}-p_{r}}{\tau }-up_{r}\delta \left(t-t_{r}\right)$, where *p*_*r*_, *τ*, *p*_0_, *u*, and *t*_*r*_ are the release probability, recovery time constant, default (maximum) release probability, depression coefficient and the release timing. At the bottom of the panel, the orange stem plot shows the timing of the release, *t*_*r*_, during an interval of 200 msec. The orange line demonstrates the release probability, *p*_*r*_, calculated from the differential equation. By assuming a time unit of Δ = 10 msec for the MRO model, the 200 msec interval corresponds to a memory of length *L* = 20. The memory content of the model is shown at the top of the panel. The blue line demonstrates the release probability calculated from the algorithm in (B). The final values of the release probability are indicated by filled circles. (D) Every arbitrary state of the release site can transit to two other states, depending on the release outcome. The transition probabilities are shown on the transition links.

We model a single release site as a binary asymmetric channel with memory (Fig 1A). The input of the channel is the presynaptic spike train, a Poisson process which is modeled by a sequence of independent Bernoulli random variables, $X={\left\{X_{i}\right\}}_{i=1}^{n}$. The random variable *X*_*i*_ corresponds to the presence (*X*_*i*_ = 1) or absence (*X*_*i*_ = 0) of the spike at time *i*, with *α* = *P*(*X*_*i*_ = 1) representing the normalized input spike rate. The output of the channel, $Y={\left\{Y_{i}\right\}}_{i=1}^{n}$, is the release outcome of the release site. If a vesicle is released at time *i*, then *Y*_*i*_ = 1 and otherwise *Y*_*i*_ = 0. The synchronous spike-evoked release mechanism of the synapse is modeled by transition from *X*_*i*_ = 1 to *Y*_*i*_ = 1, and the transition probability *p*_*i*_ represents the synchronous release probability. The asynchronous and spontaneous release modes are modeled together by transition from *X*_*i*_ = 0 to *Y*_*i*_ = 1, and *q*_*i*_ is called the asynchronous release probability.

We use a memory of the last *L* release outcomes of the channel to implement the short-term depression in our model. The release probabilities of the release site, *p*_*i*_ and *q*_*i*_, are determined by,

$$\begin{array}{c} p_{i}=p_{i}\left(Y_{i-L},Y_{i-L+1},\ldots ,Y_{i-2},Y_{i-1}\right), \end{array}$$ (1)

$$\begin{array}{c} q_{i}=q_{i}\left(Y_{i-L},Y_{i-L+1},\ldots ,Y_{i-2},Y_{i-1}\right). \end{array}$$ (2)

After each successful release, the synchronous and asynchronous release probabilities decrease to a fraction of their earlier values. This fraction is represented by the multiplier *c* or *d*, depending on the type of release. In quiescent intervals, in which no vesicle is released, the release probabilities gradually recover back to their default values (*p*_0_ and *q*_0_) with recovery coefficients *e* and *f*. The algorithm in Fig 1B describes how the synchronous release probability, *p*_*i*_, is calculated from the release site’s history (*Y*_*i*−*L*_, *Y*_*i*−*L*+1_, …, *Y*_*i*−2_, *Y*_*i*−1_). The asynchronous release probability, *q*_*i*_, is independently parameterized by the depression multiplier *d* and the recovery coefficient *f*. The interval between two discrete time indices *i* and *i* + 1 is called the time unit of the model and is represented by Δ. Throughout this paper, we set Δ = 10 msec. The biological interpretation of Δ, as well as the other model parameters, is discussed in more details in Section C of the S1 Text. Our model can reproduce the depression and recovery dynamics of the release site and is consistent with the probabilistic models of synaptic depression [22] (Fig 1C). Throughout this paper, we refer to the model as the binary asymmetric channel with a Memory of Release Outcomes, abbreviated by MRO.

To study the synaptic information efficacy of the release site under depression, we use information-theoretic measures (please see Section A of the S1 Text for an overview). The information rate of the release site is derived by calculating the mutual information between the presynaptic input spike train, *X*, and the release outcome process of the release site *Y*.

The release site in the MRO model can be in any one of 2^*L*^ states. Let *j* = *Y*_*i*−1_ + 2*Y*_*i*−2_ + 2^2^*Y*_*i*−3_ +…+ 2^*L*−1^*Y*_*i*−*L*_, 0 ≤ *j* ≤ 2^*L*^ − 1, be an arbitrary state of the release site with synchronous and asynchronous release probabilities *p*(*j*) and *q*(*j*). It can be easily shown that the mutual information rate of the binary asymmetric channel at state *j*, denoted by *R*_*j*_, is equal to

$$\begin{array}{c} R_{j}=h(\bar{\alpha }q\left(j\right)+\alpha p\left(j\right))-\bar{\alpha }h(q(j))-\alpha h(p(j)), \end{array}$$ (3)

where $\bar{\alpha }=1-\alpha$ and $h\left(x\right)=-x\;{\text{log}}_{2}\left(x\right)-\bar{x}\;{\text{log}}_{2}\left(\bar{x}\right)$.

**Fig 2:**
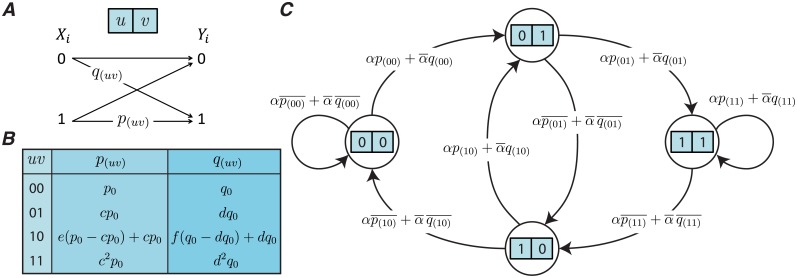
An example of the MRO model. (A) The binary asymmetric channel with a memory of length *L* = 2. (B) The table of release probabilities for every possible release outcome. The synchronous and asynchronous release probabilities are calculated from the Algorithm in Fig 1B. (C) The Markov chain model representing the transitions between the 2^*L*^ = 4 states of the model. The transition probabilities are calculated using the values in table (B) and the rule given in Fig 1D.

Each state of the release site can transit to two other states, depending on the release outcome (Fig 1D). The state transitions of the release site are modeled by a Markov chain with 2^*L*^ states (e.g., Fig 2 shows the Markov chain for the case of *L* = 2). We prove that regardless of the initial state, the probability of each state *j* converges to a stationary probability *π*_*j*_. The stationary probabilities are calculated using the power iteration method [25].

The next theorem provides a closed-form expression for the information rate of the release site.

**Theorem 1**. *Let R*_*D*_ *be the mutual information rate of the release site with short-term depression. Then*

$$\begin{array}{c} R_{D}=\sum_{j=0}^{2^{L}-1}R_{j}\pi_{j}. \end{array}$$ (4)

This theorem shows that the mutual information rate of the release site is equal to the statistical average over the information rates of its constituent states. Therefore, the rate of every release profile has a linear share in the overall information rate of the release site; the share is determined by the occurrence probability of the profile. This theorem is an extension of the result that we derived for a two-state model of depression (equivalent to *L* = 1) [24]. All the proofs are found in Section D of the S1 Text.

The brain uses more energy on synaptic transmission than on any other process [26]. To gain a better understanding of the trade-off between the energy consumption and information rate in a synapse during short-term depression, we consider the energy cost of synaptic release and derive the energy-normalized information rate of the release site. The energy-normalized information rate is calculated by dividing the mutual information (between the input and output processes) of the release site by the total amount of energy that is consumed for synaptic release. This measure quantifies the amount of information that can be transferred through the release site for one unit of energy (see Section A of the S1 Text for the mathematical formulation of these concepts).

The next theorem gives a simple expression for calculating the energy-normalized information rate of the release site.

**Theorem 2**. *Assume that the neuron consumes one unit of energy for each vesicle release. If we denote the energy-normalized information rate of the release site under depression by* $R_{D}^{\left(E\right)}$, *then*

$$\begin{array}{c} R_{D}^{\left(E\right)}=\frac{\sum_{j=0}^{2^{L}-1}R_{j}\pi_{j}}{\sum_{j=0}^{2^{\left(L-1\right)}-1}\pi_{2j+1}}. \end{array}$$ (5)

The energy normalized information rate, $R_{D}^{\left(E\right)}$, can be used to evaluate the compromise between the rate of information transfer and the energy consumption of the synapse.

We derived the mutual information rate and energy-normalized information rate of a synapse with depression in Theorem 1 and Theorem 2. For a synapse without depression, we can use the same theorems to calculate the corresponding information rates.

**Corollary 1**. *Let R*_0_ *and* $R_{0}^{\left(E\right)}$ *be the mutual information rate and energy-normalized information rate of the release site ‘without’ depression. Then*

$$\begin{array}{c} R_{0}=h\left(\bar{\alpha }q_{0}+\alpha p_{0}\right)-\bar{\alpha }h\left(q_{0}\right)-\alpha h\left(p_{0}\right), \end{array}$$ (6)

$$\begin{array}{c} R_{0}^{\left(E\right)}=\frac{R_{0}}{\bar{\alpha }q_{0}+\alpha p_{0}}. \end{array}$$ (7)

In contrast to the MRO model, for which the current state is determined by the last *L* releases, another approach would be to let the channel’s state depend only on the release outcome and the release probabilities at time *i* − 1, i.e.,

$$\begin{array}{c} p_{i}=p_{i}\left(Y_{i-1},p_{i-1}\right), \end{array}$$ (8)

$$\begin{array}{c} q_{i}=q_{i}\left(Y_{i-1},q_{i-1}\right). \end{array}$$ (9)

To compute the mutual information rate analytically for this second model, we need to quantize the release probabilities to a finite set of possible *p*_*i*_ and *q*_*i*_, as we describe in detail in Section E of the S1 Text. The two models generate similar performance results (please see Section F of the S1 Text). Table 1 gives a summary of notations used in this paper.

**Table 1:** Definition of notations.

Symbol	Definition
*X*	Presynaptic input spike process
*Y*	Release outcome process
*L*	Memory length of the release site
*p*	Synchronous spike-evoked release probability
*q*	Asynchronous release probability
*c*	Depression multiplier for synchronous spike-evoked release
*d*	Depression multiplier for asynchronous release
*e*	Recovery coefficient for synchronous spike-evoked release
*f*	Recovery coefficient for asynchronous release
*α*	Normalized input spike rate
Δ	Time unit of the MRO model
*R*_*D*_	Mutual information rate of the release site with depression
$R_{D}^{\left(E\right)}$	Energy-normalized information rate of the release site with depression
*R*_0_	Mutual information rate of the release site without depression
$R_{0}^{\left(E\right)}$	Energy-normalized information rate of the release site without depression

## Results

### Effective memory length

**Fig 3:**
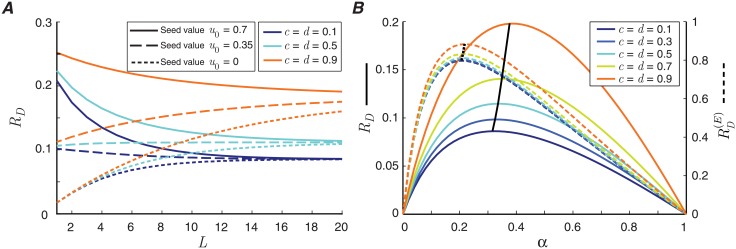
(A) The mutual information rate of the release site as a function of the memory length of the channel, *L*, for different values of depression coefficients. Three different seed values, *u*_0_, are used in the algorithm in Fig 1B to measure the effective memory of the depressing synapse. (B) The mutual information rate (solid lines), *R*_*D*_, and the energy-normalized information rate (dashed lines), $R_{D}^{\left(E\right)}$, as a function of input spike rate, *α*, for various depression coefficients, *c* and *d*. The black lines connect the maximum values of the curves. The other parameters of the model are *p*_0_ = 0.7, *q*_0_ = 0.1 and *e* = *f* = 0.1. In (A), *α* = 0.3, and in (B), *L* = 20.

Short-term synaptic depression represents a memory buffer for the synapse, as the current release dynamics of the synapse depends on the history of releases. When presynaptic spikes accumulate, the initial state of the synapse, as measured by its release probability, is slowly forgotten. We measure the effective memory length of short-term depression by calculating the time that the synapse requires to become independent from its past (which is represented by the seed value, *u*_0_, in the algorithm in Fig 1B). This effective memory length can differ from the nominal recovery time constant of the synapse from a single release. We find that, if the release probability of the synapse is halved after each release (*c* = *d* = 0.5), after 160 msec (corresponding to *L* = 16), the relative variation of the mutual information caused by different initial values drops to 10% (Fig 3A). For a synapse with stronger depression (e.g., *c* = *d* = 0.1), the effective memory of the synapse reduces to 120 msec.

The memory length of the MRO model, *L*, should match the effective memory of the synapse. We show in Section C of the S1 Text that for a large enough *L*, the mutual information rate of the MRO model converges to the information rate of a classical stochastic model of depression [22], the latter of which can only be evaluated numerically.

### Information capacity

The capacity of a release site is the maximum amount of information that can be transferred through it. We show that the capacity is reduced significantly by increasing the depression level (i.e. reducing *c* and *d*, while *c* = *d*). In the temporal coding framework, asynchronous and spontaneous release can be associated with the noise component of signal transmission, since they give rise to a postsynaptic potential in the absence of a presynaptic spike. Short-term depression reduces the release probability of asynchronous release, leading to lower noise at the release site. In contrast, as the rate of information transmission is mainly determined by the synchronous release of vesicles, depression of the synchronous release mode has a negative impact on the information rate. Therefore, if depression affects synchronous and asynchronous release equally, the overall “signal to noise” ratio decreases and information efficacy of the synapse degrades. In Section B of the S1 Text, it is shown that increasing the recovery time constant of short-term depression also deteriorates the signal-to-noise ratio.

For a weakly depressing synapse with high synchronous release probability, the corresponding communication channel is akin to an ideal channel. Hence the optimal spike rate is close to the rate *α* = 0.5, which yields a spike train with maximal entropy. As the level of short-term depression increases, the communication channel becomes more unreliable and the uncertainty of the release outcome *Y*_*i*_ given an input spike *X*_*i*_ = 1 increases. Given that synaptic depression penalizes higher input spike rates, the capacity (maximal information rate) is attained at lower input rates (solid lines in Fig 3B).

### Energy-normalized information rate

The total energy consumption of the synapse is determined by the number of releases, so it is a monotonically increasing function of the input spike rate and the release probability. Increasing the level of short-term depression reduces both the mutual information rate and the energy consumption of the synapse. As both quantities decrease in equal measure under depression, the ratio of mutual information to total energy expenditure, which defines the energy-normalized information rate, is rendered robust to variations in the parameters of depression, as long as the input spike rate is fixed. As a corollary, the spike rate that optimizes the release site’s rate-energy trade-off is independent of the depression level and its associated recovery constant (dashed lines in Fig 3B). Our numerical analysis shows that even synapses with different synaptic dynamics ought to be activated at similar rates to work optimally (see also Section B of the S1 Text).

### Critical ratio

**Fig 4:**
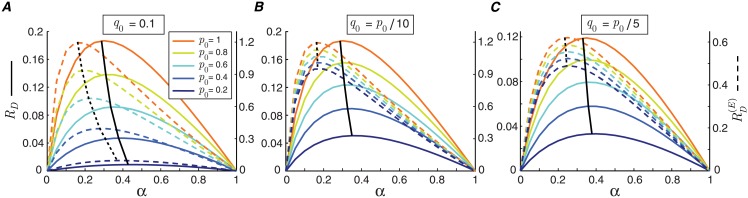
The ratio between synchronous and asynchronous release probability controls the optimal operating point of the synapse. Mutual information rate (solid lines) and energy-normalized information rate (dashed lines) of the synapse are plotted as a function of input spike rate for different values of default (maximum) synchronous spike-evoked release probability, *p*_0_. (A) The default asynchronous release probability is fixed at *q*_0_ = 0.1. The black lines connect the maximum values of the curves. (B) The default asynchronous release probability is a fraction of the default synchronous spike-evoked release probability, *q*_0_ = *p*_0_/10. (C) Similar to (B) for *q*_0_ = *p*_0_/5. The other parameters of the model are *L* = 20, *c* = *d* = 0.5, and *e* = *f* = 0.1.

The mutual information rate of a synapse changes substantially with the synchronous release probability *p*_0_ (Fig 4A). Provided that the ratio between synchronous and asynchronous release probability remains constant ($\frac{q_{0}}{p_{0}}=K$), then dividing the mutual information rate by the energy consumption of the synapse largely eliminates the dependency of mutual information rate on *p*_0_. Consequently, the energy-normalized information rate and optimal input spike rate are nearly independent of the release probability *p*_0_ (Fig 4B and 4C).

### State-space representation of a synapse

**Fig 5:**
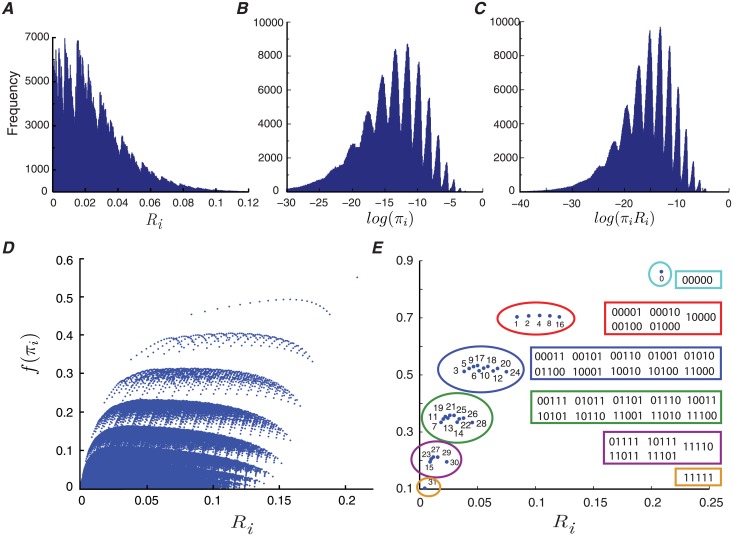
Distribution of the release site’s states for *L* = 20. (A) Histogram of the mutual information rates of the states, *R*_*i*_. (B) Histogram of the logarithm of states’ stationary probabilities, log(*π*_*i*_). The logarithm function shows the dynamic range of probabilities more clearly. (C) The histogram of log(*R*_*i*_ *π*_*i*_). (D) The stationary probability of each state is plotted against its mutual information rate. The clusters get more prominent by setting *f*(*x*) = *x*^0.15^. (E) Similar to (D), for a release site with a memory length of *L* = 5. The corresponding memory pattern is also shown for each state. The other simulation parameters are *c* = *d* = 0.5, *e* = *f* = 0.1, *p*_0_ = 0.7, *q*_0_ = 0.1, and *α* = 0.2.

A release site with a memory length of *L* = 20 consists of more than one million states. In Theorem 1, we prove that the mutual information rate of the release site is equal to the statistical average of the information rates of its constituent states. Therefore, the distribution of information rates and stationary probabilities of the states specifies the share of the memory patterns in the mutual information rate. We show that there are no dominant states for the release site. Indeed, the majority of the states have a very low mutual information rate (Fig 5A). We also calculate the distribution of stationary probabilities (Fig 5B) and the distribution of the product of rates and stationary probabilities of the states (Fig 5C). The states of the release site cluster, as seen in the rate-probability representation in Fig 5D. To characterize the clusters, we identify them for the case of *L* = 5 (Fig 5E). The clusters each turn out to represent a fixed number of releases within the release site’s history.

### Rate-energy classes of synapses

**Fig 6:**
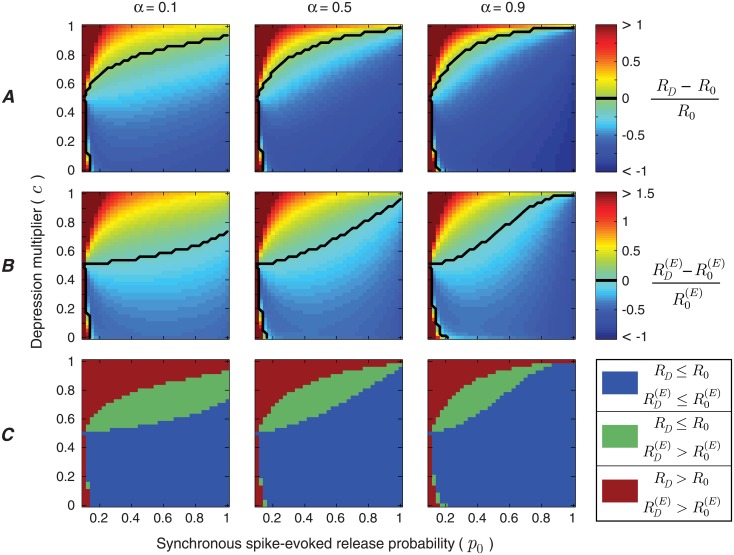
The impact of synchronous spike-evoked release dynamics on synaptic information transmission during short-term depression. (A) Relative difference between the mutual information rates of the release site with and without depression, as a function of depression multiplier, *c*, and release probability, *p*_0_, of synchronous spike-evoked release. The black line indicates the boundary at which the functional role of synaptic depression switches from degradation to enhancement of information rate. Each column corresponds to a different input spike rate. (B) Relative difference between the energy-normalized information rates of the release site with and without depression under the same conditions as (A). (C) Classification of release sites to three functional categories based on the impact of depression on the mutual information rate and energy-normalized information rate of the release site. The parameters of asynchronous release are fixed at *d* = 0.5 and *q*_0_ = 0.1, and the recovery coefficients are *e* = *f* = 0.1.

We now study how depression dynamics of synchronous spike-evoked release affect the information efficacy of the release site, while keeping the dynamics of asynchronous release fixed. We show that for low values of synchronous release probability, *p*_0_, and high values of synchronous spike-evoked depression multiplier, *c*, short-term depression increases the mutual information rate (Fig 6A) and energy-normalized information rate (Fig 6B) of the release site. When the asynchronous release (which is associated with the noise in release) depresses more than the synchronous release (which is associated with the signal component of release), the overall “signal to noise” ratio of the release site can be enhanced by short-term depression. However, if the synchronous release probability is much higher than asynchronous release probability (i.e., *p*_0_ ≫ *q*_0_), even a slight depression of synchronous release lowers the “signal to noise” ratio remarkably and as a result, the information rate decreases during short-term depression.

Under unusual circumstances, stronger synaptic depression of synchronous release can improve the information rate. Such a situation arises when the synapse as a communication channel inverts the input spike train, which can happen when the initial release probability *p*_0_ is very low. In that case, stronger depression of synchronous release enhances the inversion of the incoming spike train.

Based on our analysis, release sites can be classified into three functional categories depending on their depression dynamics (Fig 6C):

Category 1: Depression increases both the mutual information rate and energy-normalized information rate of the release site.
Category 2: Depression increases the energy-normalized information rate, while the mutual information rate of the release site is reduced.
Category 3: Depression impairs the performance of the release site by decreasing both the mutual information rate and the energy-normalized information rate.

The enhancement effect of depression on the synaptic information efficacy is larger for the synapses with lower input spike rates, because the impact of asynchronous release is more significant at lower input spike rates. Also, the three categories imply that the enhancement of energy-normalized information rate is a necessary condition for the increase of mutual information rate during depression. We also note that the recovery coefficient of synchronous spike-evoked release has a similar impact on the synaptic information efficacy and creates the same functional categories (refer to Section B of the S1 Text).

### Critical role of asynchronous release

**Fig 7:**
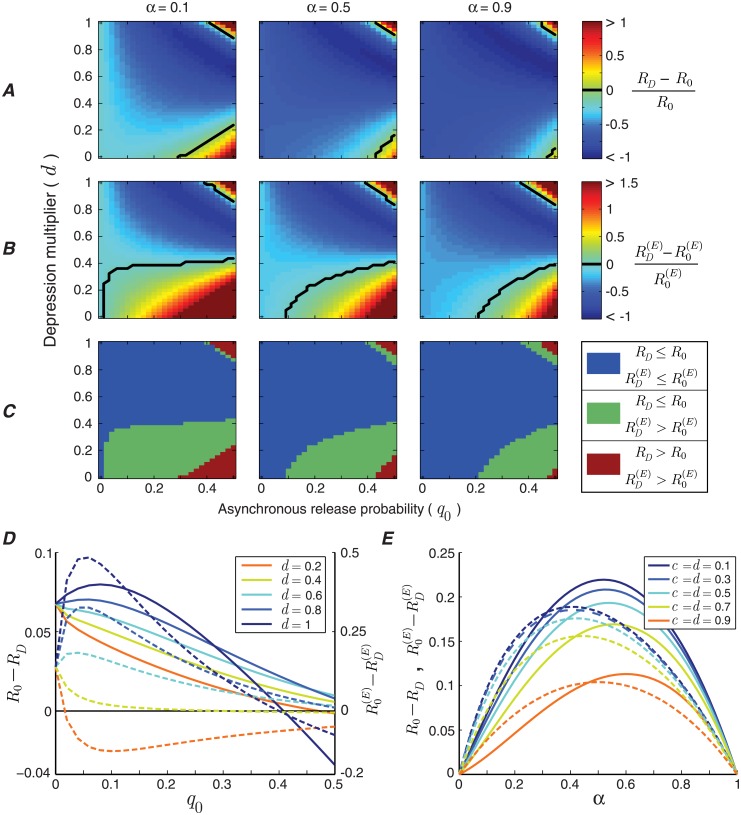
The modulatory effect of asynchronous release dynamics on synaptic information efficacy during short-term depression. (A) Relative difference between the mutual information rates of the release site with and without depression, as a function of depression multiplier, *d*, and release probability, *q*_0_, of asynchronous release. For the synchronous spike-evoked release, the dynamics is kept fixed at *c* = 0.5 and *p*_0_ = 0.7. The black line shows the boundary between positive and negative values. (B) Relative difference between the energy-normalized information rates of the release site with and without depression. (C) Three functional categories of the release site during depression. (D) Changes in the mutual information rate (solid lines), *R*_0_−*R*_*D*_, and energy-normalized information rate (dashed lines), $R_{0}^{\left(E\right)}-R_{D}^{\left(E\right)}$, during depression. The functions are plotted against asynchronous release probability, *q*_0_, for different values of depression multiplier, *d*. (E) The impact of similar depression dynamics for asynchronous and synchronous spike-evoked release (*c* = *d*) on the rate changes during depression. We simulate *R*_0_ − *R*_*D*_ and $R_{0}^{\left(E\right)}-R_{D}^{\left(E\right)}$ as a function of input spike rate, *α*, for different values of depression multiplier. For the simulations in this figure, the recovery time constants are fixed at *e* = *f* = 0.1.

Although asynchronous and spontaneous releases are usually ignored in information rate analysis, we show that their dynamics have a critical impact on the synaptic information efficacy during short-term depression; the release probability and depression multiplier of asynchronous release can completely change the regime of information transmission (Fig 7A–7C). We see that for synapses with larger asynchronous release probability, *q*_0_, and lower depression multiplier, *d*, the mutual information rate and energy-normalized information rate increase during short-term depression. On the other hand, in the absence of asynchronous release (*q*_0_ = 0), depression always decreases both the mutual information rate and energy-normalized information rate of the release site (Fig 7D). Interestingly, if asynchronous release does not depress at all (*d* = 1), depression can still increase the information rate of the release site, provided that asynchronous release probability, *q*_0_, is large enough (Fig 7D). In addition, as long as asynchronous and synchronous spike-evoked release have similar depression dynamics (*c* = *d* and *e* = *f*), depression will always decrease the energy-normalized information rate (Fig 7E).

### Information efficacy of experimentally measured synapses

Here we use the experimental measurements of three synapses and assess the information efficacy of each synapse during short-term depression.

#### Hippocampal synapses

In the hippocampal autaptic synapses, synchronous spike-evoked release and asynchronous release compete for a common limited pool of vesicles [27]. Although synchronous release is attenuated by short-term depression, the asynchronous component of release is not affected; instead the rate of asynchronous release is preserved. It has been suggested that asynchronous release can compensate for the depression of synchronous release, so that the rate of information transmission will remain unaffected [27]. Here, we apply our framework to assess this prediction quantitatively.

We fit the depression dynamics of synchronous release to Fig. 1Db of [27] and estimate the depression multiplier of synchronous spike-evoked release as *c* = 0.75. This value is also consistent with the paired-pulse measurements of EPSP in the same type of synapse where the ratio of the second EPSP to the first EPSP is 0.78 ± 0.04 [28]. The default (maximum) synchronous spike-evoked release probability is *p*_0_ = 0.4, and the actual recovery time constant of synchronous release is suggested to be 250 msec (corresponding to *e* = 0.039 in our model) [27].

In hippocampal synapses, the stationary value of asynchronous release probability increases with the input spike rate, however, it is not affected by short-term depression (i.e., *d* = 1) [29]. We estimate asynchronous release probabilities at input spike rates of 5 Hz, 10 Hz, and 20 Hz using the asynchronous release ratios in Fig. 5A of [29]. We use a base value for the asynchronous release probability of 0.002/msec, as derived from measurements in a single stimulation protocol [30]. Translating these parameters into our framework, we have *q*_0_(*α* = 5 Hz) = 0.04, *q*_0_(*α* = 10 Hz) = 0.08, and *q*_0_(*α* = 20 Hz) = 0.12.

Using Theorems 1 and 2, we estimate the mutual information rate (in bits per second: bps) and energy-normalized information rate (in bits per second per unit of energy: bps/*E*) of the hippocampal synapse with and without depression for different input spike rates (Table 2). In contrast to the conclusion in [27], our analysis shows that asynchronous release in hippocampal synapses does not preserve the temporally encoded information about the presynaptic spike train during short-term depression. Synaptic depression degrades both the information rate and the energy-normalized rate.

**Table 2:** The impact of short-term depression on the mutual information rate and energy-normalized information rate of hippocampal synapses.

Input spike rate (Hz)	*R*_0_ (bps)	*R*_*D*_ (bps)	$R_{0}^{\left(E\right)}$ (bps/*E*)	$R_{D}^{\left(E\right)}$ (bps/*E*)
5	4.1	3.0	70.2	54.6
10	4.7	2.3	41.8	22.9
20	5.3	1.4	30.4	10.1

#### Calyx of Held

The calyx of Held, a well-studied synapse in the mammalian nervous system, contains both fast phasic release and slow asynchronous release [31–33]. Notably, the time-course of recovery of synaptic release for these two release components, as measured experimentally, is very different: around 200 milliseconds for asynchronous release, and up to 4.2 seconds for spike-evoked synchronous release (as reviewed in [31, 34]). The strength of synaptic depression has been measured by fitting time constants to the apparent decay of averaged postsynaptic voltage responses. The corresponding decay time constant of synchronous release is 159 msec (for a 10 Hz stimulation) [34], while asynchronous release decays with a much shorter time constant of 15 msec [32]. This discrepancy in time scales accentuates the importance of considering the potential differences between the depression dynamics of synchronous and asynchronous release in computational models of synaptic transmission, as we did in this study.

The maximum (default) synchronous spike-evoked release probability at the calyx of Held is between 0.25 to 0.4 [35]. Measurements in the calyx of Held indicate that the rate of asynchronous release is highly variable among synapses; the release rates between 0.2 to 15.2 vesicles per millisecond (with an average of 2.3 vesicles per millisecond) have been observed [32].

We now study the impact of the broad range of asynchronous release rates on the information efficacy of release sites in the calyx of Held. The parameters of the model are estimated using the aforementioned measurements: *c* = 0.53, *d* = 0.5, *e* = 0.0024, *f* = 0.0153, *p*_0_(*average*) = 0.32, *a* = 0.1, *q*_0_(*minimum*) = 0.0033, *q*_0_(*average*) = 0.038, and *q*_0_(*maximum*) = 0.25. To estimate these parameters, we have assumed the calyx of Held has on average 600 active zones [9, 35–37]. The time unit of the model is Δ = 10 msec.

Table 3 displays the mutual information rate (in bits per second: bps) and energy-normalized information rate (in bits per second per unit of energy: bps/*E*) of a single release site in the calyx of Held for the minimum, average, and the maximum of asynchronous release probability. The results reveal that asynchronous release remarkably degrades the information efficacy of synaptic transmission in the calyx of Held; it drops nearly to zero for synapses with very high asynchronous release rates.

**Table 3:** Information efficacy of a single release site in the calyx of Held during short-term depression.

Asynchronous release probability	*R*_0_ (bps)	*R*_*D*_ (bps)	$R_{0}^{\left(E\right)}$ (bps/*E*)	$R_{D}^{\left(E\right)}$ (bps/*E*)
0.0033 (minimum)	9.9	7.6	284.6	280.5
0.038 (average)	5.1	3.2	77.6	72.9
0.25 (maximum)	0.16	0.0003	0.6	0.3

The level of depression for asynchronous release is higher than the depression level of synchronous release, however, asynchronous release recovers much faster. The competition between the depression dynamics of synchronous and asynchronous release leads to a decline in mutual information rate of the synapse with depression. Nevertheless, the energy-normalized information rate remains almost unchanged, suggesting that under metaplasticity, the rate of information per release could be preserved in the calyx of Held.

#### Corticostriatal synapses in nucleus accumbens

Measurements in nucleus accumbens reveal asynchronous vesicular release in corticostriatal synapses [38]. The synapse is stimulated by 8 stimuli with a frequency of 25 Hz, and the depression dynamics of synchronous release and the rate of asynchronous release are estimated [38, 39]. The recovery time constant of synchronous release is 406 msec (corresponding to *e* = 0.024), and the depression multiplier of synchronous spike-evoked release is estimated as *c* = 0.75 by fitting the depression dynamics to the EPSP responses of the eight stimuli (Fig. 1 in [39]). Also the probability of synchronous spike-evoked release has been measured as *p*_0_ = 0.42 [40].

Asynchronous release comprises 40% of the releases that occur in the first 20 msec after the eighth stimulation. Since the synchronous release probability of the eighth stimulus is approximately 0.28 × *p*_0_, the probability of asynchronous release would be *q*_0_ = 0.04 (for a time unit of Δ = 10 msec). It is also assumed that asynchronous release is not depressed in corticostriatal synapses, i.e., *d* = 1.

Dopamine modulates the activity of the medium spiny neurons in nucleus accumbens by inhibiting the release of glutamate and GABA neurotransmitters [39, 41]. In experiments, application of dopamine (75 *μM*) reduces the rate of asynchronous release by 46% (corresponding to *q*_0_ = 0.01), but does not affect the steady-state rate of synchronous release [39].

We use our framework to study the modulatory impact of dopamine on the information efficacy of corticostriatal synapses during short-term depression. In Table 4, we compare the mutual information rate and energy-normalized information rate of the afferent excitatory synapses at medium spiny neurons in nucleus accumbens in the presence or absence of dopamine. Our analysis shows that the release of dopamine increases the information efficacy of corticostriatal synapses, allowing the nucleus accumbens to receive more information from cortical afferent fibers.

**Table 4:** Dopamine modulates information efficacy of corticostriatal synapses during short-term depression.

Dopamine (*μM*)	*R*_*D*_ (bps)	$R_{D}^{\left(E\right)}$ (bps/*E*)
0	7.0	72.2
75	10.6	125.7

## Discussion

By modeling a single synaptic release site as a binary asymmetric channel with memory, we were able to derive the information rate of synaptic release analytically. Such theoretical models rely on quantization, but the theoretical results are fully consistent with the numerical evaluation of experimentally motivated stochastic models of short-term depression [22, 42]. The MRO model presented here is an extension of a two-state model of depression [24]. By incorporating multiple states, the MRO model can capture the gradual depression and recovery of synapses more precisely.

In contrast to many other approaches, our calculations are not limited to synchronous spike-evoked release, as they also treat asynchronous and spontaneous releases. Asynchronous release can occur from tens of milliseconds to tens of seconds after the arrival of an action potential. It depends on the intracellular concentration of calcium and is mediated by specialized calcium sensors with slow kinetics [3]. Spontaneous release, on the other hand, occurs in the absence of an action potential by fluctuations in the resting concentration of calcium or stochastic opening of calcium channels [4, 43]. It is presumed that spontaneous release is elicited by the same calcium sensor as asynchronous release [44, 45]. Therefore, in this study, we subsumed spontaneous vesicular release into the category of asynchronous release. In contrast, the depression dynamics of synchronous release and asynchronous release (including spontaneous release) could well differ, as they depend on distinct calcium signaling pathways and distinct SNARE proteins as part of the synaptic release machinery [3, 4, 46–50]. To take these structural and functional differences into account, we assigned distinct set of parameters to depression dynamics of synchronous and asynchronous release.

Strikingly, we were able to show that synaptic depression can *enhance* information transmission provided that synchronous spike-evoked release is depressed less (or recovers faster) than the asynchronous release. On the other hand, if the depression dynamics for both synchronous and asynchronous release are the same, then synaptic depression always decreases the information rate of the release site, as we proved. Short-term plasticity differs widely in its dynamics across synapses [51]. Our results, therefore, suggest that synapses fall into one of three functional categories, based on the relative effects of depression on synchronous spike-evoked release and asynchronous release (Fig 6C): depression can be deleterious, can improve the energy-normalized information rate, or even improve the overall information rate.

We proved that the overall information rate is the linear sum of the information rates for every release-history-dependent state, weighted by the stationary probability of being in that state. The simplicity of this result is non-trivial; under short-term facilitation, for instance, it can be shown that the information rate is no longer a statistical average over states.

Synaptic release is energetically expensive [26, 52, 53]. Indeed, it has been hypothesized that synaptic mechanisms optimize the energy-information rate balance during neuronal transmission [15, 26, 54]. To study the energy-information trade-off at the release site, we calculated the energy-normalized information rate analytically. Only the energy that is consumed by synaptic release was taken into account, which ignored the energy expenditure needed for the generation of action potentials, cellular homeostasis, or protein synthesis and transport.

In comparison to the information rate, the energy-normalized information rate of the release site was much more robust to variations in the depression dynamics. Specifically, the optimal presynaptic spike rate was invariant. The spike rate needed to achieve information capacity, in contrast, was sensitive to the strength of depression, as stronger depression implied lower input spike rates. Notably, the depression dynamics vary across synapses and release sites, even in the same neuron [5]. Metaplasticity changes the depression characteristics of the release site over different time scales [55, 56]. The prediction of our work is that the input spike rate is uncoupled from synaptic metaplasticity: the input rate need not adapt to maintain the optimal energy-information balance for release sites.

In [21, 57], it is shown that short-term depression can increase the rate of information transmission, provided that the input spike process is correlated; if the incoming spike train is Poisson, short-term depression reduces both the mutual information [21] and the Fisher information [57]. In contrast to these studies, we show that by considering the other modes of release (i.e. asynchronous and spontaneous release), short-term depression can enhance the rate of information transmission in the synapse, even for Poisson inputs. Our results demonstrate the importance of asynchronous and spontaneous release in synaptic information transmission and indicate that the inclusion of the other modes of release can completely switch the functional role of short-term depression. If the release mode of a synapse is confined to synchronous spike-evoked release (i.e. *q*_0_ = 0), our analysis replicates the results of the previous studies. However, for a synapse with significant asynchronous or spontaneous release, the results in [21, 57] no longer necessarily hold, and our framework can be employed, instead, to calculate synaptic information efficacy.

Our calculations presume that there is a single pool of vesicles for synchronous, asynchronous, and spontaneous release. Data from several studies suggest that vesicles released synchronously and asynchronously come from the same pool of vesicles [3, 27, 58, 59]. Whether the same pool supplies vesicles for spontaneous release is a matter of considerable debate, as a number of studies argue that spontaneous release uses a distinct pool of vesicles [59–61], while others argue the opposite [62–64]. The existence of independent vesicle pools would change synaptic information transfer; how such a scenario could still be incorporated into our mathematical framework is explored in Section G of the S1 Text.

We employed our framework to study information efficacy of three types of synapses using available experimental measurements. In hippocampal synapses, we showed that in contrast to an earlier suggestion [27], asynchronous release fails to compensate for the depression of synchronous release; both the mutual information rate and the energy-normalized rate of the synapse decrease during short-term depression. This result holds true for the temporally encoded information in the spike train. This finding does not rule out that asynchronous release could help sustain the synaptically transferred information about changes in the temporally coarse-grained presynaptic firing rate; we only considered binary spike trains for which the timing of each spike counts.

Measurements in the calyx of Held reveal distinct recovery time constants for synchronous and asynchronous releases [31]. Our computational model of synaptic transmission permits dissimilar depression dynamics, so we could use the calyx of Held as another test case. We found that asynchronous release strongly affects the information efficacy of the calyx of Held with or without synaptic depression. Moreover, we discovered that were synaptic depression to change in strength in the calyx of Held, the energy-normalized information rate would not be greatly upset, which opens up the possibility that energy use in the calyx of Held is regulated through metaplasticity of short-term depression. We also studied the modulatory role of dopamine in corticostriatal synapses of nucleus accumbens. Our analysis revealed that dopamine increases the information rate of the synapses in nucleus accumbens by presynaptic inhibition of asynchronous release.

We now list a few of the limitations of the model. Strictly speaking, the proposed model is valid for a single synaptic release site. The number of release sites in a synapse varies between one to hundreds, with most central nervous system synapses having one or two sites [65]. Some studies have addressed the information efficacy of the whole synapse by treating all the release sites similarly [18, 66], neglecting the individual differences of the release sites [35]. It should be possible to use parallel MRO models (with potentially distinct dynamics) to calculate the information rate of the entire synapse.

We only considered constant-rate input spike trains here. However, synaptic depression not only shapes the synaptic information channels, but directly implements temporal filtering, making neurons more sensitive to changes in presynaptic rate rather than the steady-state rate [1, 11, 12, 51, 67]. The input model can be generalized to heterogeneous Poisson processes to account for the rate changes of the input in the presence or absence of a stimulus.

To completely resolve the puzzle of information transmission between two neurons, we would still need to consider the feedback mechanisms of the synapse [68, 69], the non-linearity of receptors at the postsynaptic neuron [1, 70, 71], and other short-term and long-term dynamical mechanisms of the synapse [5, 72]. Filling in these gaps will yield a complete picture of synaptic information transmission. We believe that the MRO model can serve as an elemental building block to develop more detailed models and aid in future research to complete the full picture of synaptic information transmission.

## Supporting information

S1 Text Supplementary material. (A) Information theoretic measures, (B) Recovery time constant and synaptic information efficacy, (C) Model parameters, (D) Proof of theorems, (E) Quantized release probabilities, (F) Comparison between the two models of short-term depression, (G) Distinct pools of vesicles.
(PDF)
