# Supplementary material for: Short-term synaptic depression can increase the rate of information transfer at a release site
Source: PLoS Comput Biol. 2019 Jan 2;15(1):e1006666. doi: 10.1371/journal.pcbi.1006666 (PMC6355030; doi:10.1371/journal.pcbi.1006666)
Supplement: S1 Text — (A) Information theoretic measures, (B) Recovery time constant and synaptic information efficacy, (C) Model parameters, (D) Proof of theorems, (E) Quantized release probabilities, (F) Comparison between the two models of short-term depression, (G) Distinct pools of vesicles. (PDF) [file pcbi.1006666.s001.pdf]

Supplementary Material

Short-term synaptic depression can increase the rate of information transfer at a release site

Mehrdad Salmasi, Alex Loebel, Stefan Glasauer, Martin Stemmler

A. Information theoretic measures

Let X be a discrete random variable with a finite sample space $\{x_1, x_2, ..., x_n\}$. The entropy of X, denoted by $H(X)$, is the amount of uncertainty about the value of X and is calculated by

$$H(X) = -\sum_i P(X = x_i) \log_2 P(X = x_i), \tag{1}$$

where $P(.)$ is the probability measure.

For two discrete random variables X and Y, the conditional entropy of Y given X, denoted by $H(Y|X)$, describes the remaining uncertainty about the value of Y provided that the value of X is known. The conditional entropy is derived from

$$H(Y|X) = -\sum_x \sum_y P(X = x, Y = y) \log_2 P(Y = y|X = x). \tag{2}$$

The mutual information between the two random variables X and Y is defined by

$$I(X;Y) = H(Y) - H(Y|X), \tag{3}$$

and quantifies the amount of information that can be obtained from X about Y.

The notions of entropy and mutual information are extended to random processes as well. Let $X = \{X_i\}_{i=1}^{\infty}$ be a discrete time random process, where X_i is the random variable corresponding to the value of X at time i. We represent by X^n the first n instances of X,

$$X^n \triangleq \begin{cases} (X_1, X_2, ..., X_n) & \text{if} \quad n > 0 \\ 0 & \text{if} \quad n \leq 0 \end{cases} \tag{4}$$

The entropy rate of X is defined by

$$H(X) = \lim_{n\to\infty} \frac{1}{n} H(X^n), \tag{5}$$

if the limit exists.

The mutual information rate of two random processes $X = \{X_i\}_{i=1}^{\infty}$ and $Y = \{Y_i\}_{i=1}^{\infty}$ is

$$I(X;Y) = \lim_{n\to\infty} \frac{1}{n} I(X^n; Y^n), \tag{6}$$

provided that the limit exists.

Assume that the random processes X and Y are the input and output of a communication channel. Let E_i be the (random) amount of energy that is consumed by the channel at time i. The energy-normalized information rate of the channel is defined by

$$I_E(X;Y) = \lim_{n\to\infty} \frac{I(X^n; Y^n)}{\mathbf{E}(\sum_{i=1}^{n} E_i)}, \tag{7}$$

where $\mathbf{E}(.)$ is the expected value.

B. Recovery time constant and synaptic information efficacy

The speed of recovery from depression modulates the rate of information transfer at a release site. Slower recovery expands the impact range of the release history and consequently, increases the effective memory length of the release site (Fig. S1A). For a synapse with a recovery time constant of $\tau = 100$ msec (corresponding to $e = 0.1$), the relative variation of the mutual information rate caused by different initial states (various seed values u_0 in the algorithm in Fig. 1B) reduces to 10% after 160 msec. The effective memory length is reduced to 70 msec for a synapse with faster recovery, τ=28 msec (equivalent to $e = 0.3$).

Faster recovery increases both the mutual information rate and energy-normalized information rate of the release site. (Fig. S1B). The mutual information rate changes substantially by the variations of recovery coefficient while the energy-normalized information rate is relatively robust. Specifically, by increasing the recovery coefficient, the capacity of the release site is attained at higher input spike rates. But the input spike rate that results in the optimal energy-normalized information rate is practically independent of the recovery time constant. From Fig. S1B and Fig. 3B, we conclude that the release sites with different depression dynamics can work at their optimal energy-rate regime with the same input spike rate.

If the recovery of the synchronous spike-evoked release is faster than the recovery of asynchronous release, then depression can increase the mutual information rate (Fig. S1C) and energy-normalized information rate of the release site (Fig. S1D). We show that the differences in recovery coefficient among synapses (and release sites) create three distinct functional categories for short-term depression (Fig. S1E).

C. Model parameters

We set the parameters of the MRO model by establishing a correspondence with an updated version of the stochastic model of depression [1]. The release probability (synchronous or asynchronous) follows a first-order differential equation in the stochastic model,

$$\frac{dp_r}{dt} = \frac{p_0 - p_r}{\tau} - up_r\delta(t - t_r), \tag{8}$$

where $p_r, \tau, p_0, u,$ and t_r are the release probability, recovery time constant, default (maximum) release probability, depression coefficient and the release timing.

In the absence of release, the release probability recovers exponentially to its default value. Assuming that the release probability at time $t = 0$ is p_{in},

$$p_r(t) = p_0 - (p_0 - p_{in})exp(-t/\tau). \tag{9}$$

Correspondingly, if we assume that in the MRO model, the release probability at time index $i = 0$ is p_{in}, then it can be easily shown that after k steps of recovery (k successive quiescent intervals),

$$p_r(k) = p_0 - (p_0 - p_{in})(1 - e)^k, \tag{10}$$

where e is the recovery coefficient of the MRO model.

The discrete time k and the continuous time t are related through the time unit, Δ, of the MRO model,

$$k = t/\Delta. \tag{11}$$

To have similar recovery dynamics in the two models,

$$(1 - e)^k = exp(-t/\tau), \tag{12}$$

and by substituting k from (11),

$$ln(1 - e) = \frac{-\Delta}{\tau}. \tag{13}$$

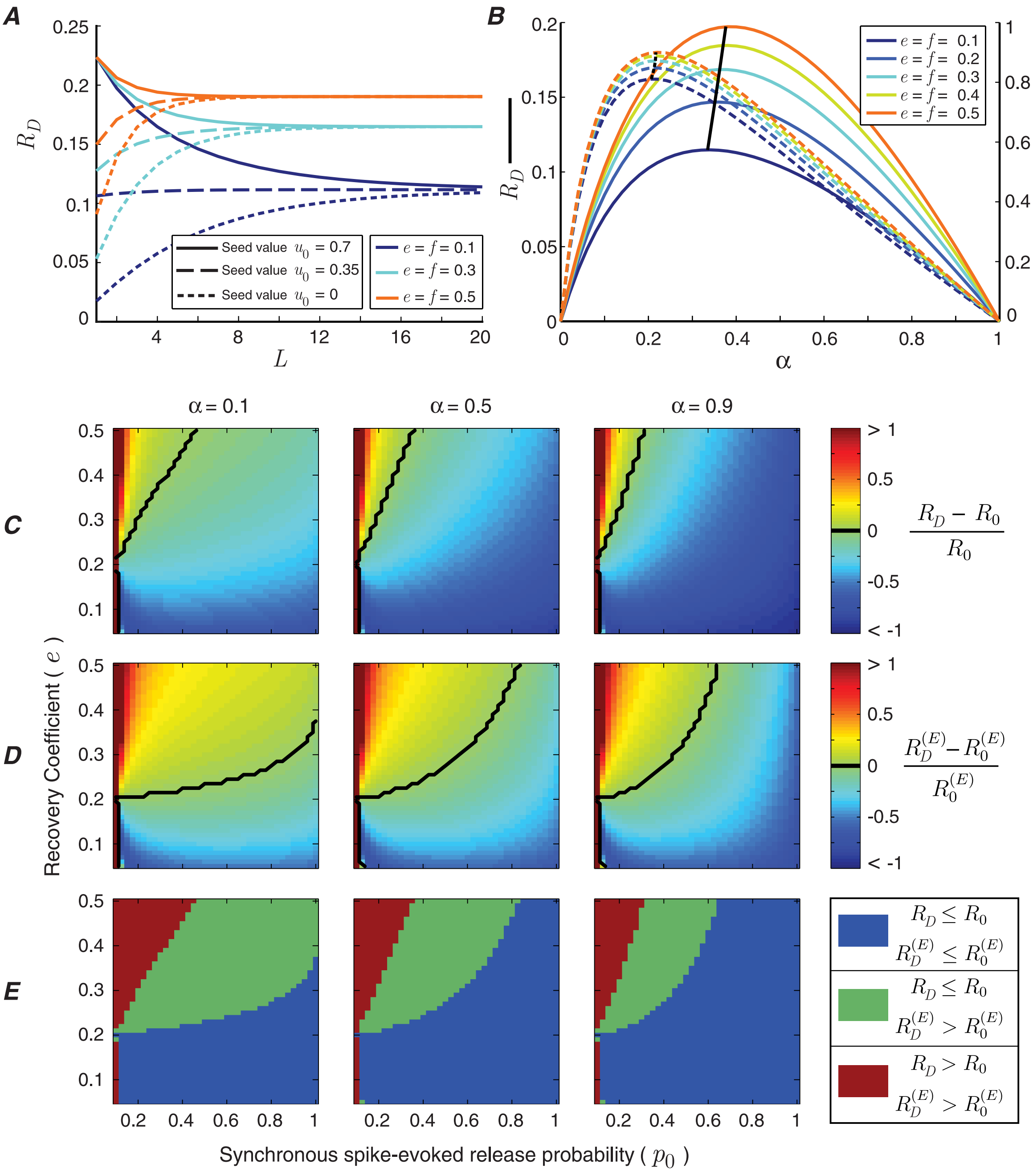

Figure S1: Modulation of information rate by the recovery coefficient of the release site. (A) Mutual information rate as a function of the release site's memory length L, for different recovery coefficients. Three different seed values, u_0, are used in the algorithm in Fig. 1B to measure the effective memory length of synaptic depression. (B) Mutual information rate (solid lines) and energy-normalized information rate (dashed lines) of the release site against the input spike rate. The black lines connect the maximum of the curves. (C) Relative difference between R_D and R_0 as a function of the recovery coefficient, e, and the release probability, p_0, of synchronous spike-evoked release. The recovery coefficient of asynchronous release is fixed at $f = 0.2$. The black lines specify the zero transitions and columns correspond to different input spike rates. (D) Relative difference between $R_D^{(E)}$ and $R_0^{(E)}$, under the same conditions as (C). (E) Three functional categories for short-term depression at a release site, determined by e and p_0. In these simulations, the depression multipliers are fixed at $c = d = 0.5$ and the default asynchronous release probability is $q_0 = 0.1$. Also the memory length is set to $L = 20$ (except in (A)). The default synchronous spike-evoked release probability is $p_0 = 0.7$ in (A) and (B), and the input spike rate is $\alpha = 0.3$ in (A).

This equation shows the relationship between the recovery coefficient of the MRO model and the recovery time constant of the synapse. For example, if the recovery time constant of a synapse is $\tau = 100$ msec, then for a time unit of $\Delta = 10$ msec, the recovery coefficient of the MRO model should be $e = 0.1$.

After a release at time t_r, the release probability of the synapse (described by (8)) decreases to

$$p_r(t_r^+) = (1-u)p_r(t_r^-). \tag{14}$$

Therefore, the depression multiplier of the MRO model, c, can be derived from

$$c = 1 - u. \tag{15}$$

However, estimation of c from (15) leads to an estimation bias, due to the slight recovery of release probability during a single time bin. Using the approximation

$$p_r(t_r^+ + \Delta) - p_r(t_r^+) = \frac{\Delta}{\tau}(p_0 - p_r(t_r^+)), \tag{16}$$

and assuming a uniform distribution for p_r, we can derive a more accurate estimation for the depression multiplier:

$$c = \frac{2\Delta}{\tau} + (1 - \frac{\Delta}{\tau})(1-u). \tag{17}$$

The memory length, L, of the MRO model represents the number of previous release outcomes that are used to determine the current release probability of the synapse. We define the effective memory length of a synapse, L_{eff}, as the minimum value of L for which the mutual information rate of the synapse becomes independent from its past (characterized by the seed value in the algorithm in Fig. 1B). We can find the effective memory of a synapse (in milliseconds) by $L_{eff} \times \Delta$. For example, if the recovery time constant of a synapse is 100 msec ($e = 0.1$) and its depression coefficient is $u = 0.67$ ($c = 0.5$), then the effective memory of the synapse is approximately 160 msec (corresponding to $L_{eff} = 16$).

We use the context tree weighting algorithm [2] to calculate numerically the information rate of the synapse in a classical, stochastic model of depression [1]. In Fig. S2, we show that by increasing the memory length, L, the analytical mutual information rate of the MRO model converges to the numerical information rate estimates of the classical stochastic model of depression.

D. Proof of Theorems

Proof of Theorem 1: By definition

$$R_D = \lim_{n\to\infty} \frac{1}{n} I(X^n; Y^n), \tag{18}$$

where

$$I(X^n; Y^n) = H(Y^n) - H(Y^n|X^n). \tag{19}$$

Using the chain rule [3],

$$H(Y^n) = \sum_{i=1}^{n} H(Y_i|Y^{i-1}). \tag{20}$$

For integer values a, b, we define

$$Y_a^b \triangleq \begin{cases} (Y_a, Y_{a+1}, ..., Y_b) & \text{if} \quad 1 \le a \le b \\ (Y_1, Y_2, ..., Y_b) & \text{if} \quad a \le 0 < b \\ 0 & \text{if} \quad a \le b \le 0 \end{cases} \tag{21}$$

The random variable Y_i depends on the release probabilities at time i, p_i and q_i, and the input spike variable at time i, X_i. Since p_i and q_i are functions of $(Y_{i-L}, Y_{i-L+1}, ..., Y_{i-1})$, and X_i is independent of Y^{i-1},

$$H(Y_i|Y^{i-1}) = H(Y_i|Y_{i-L}^{i-1}). \tag{22}$$

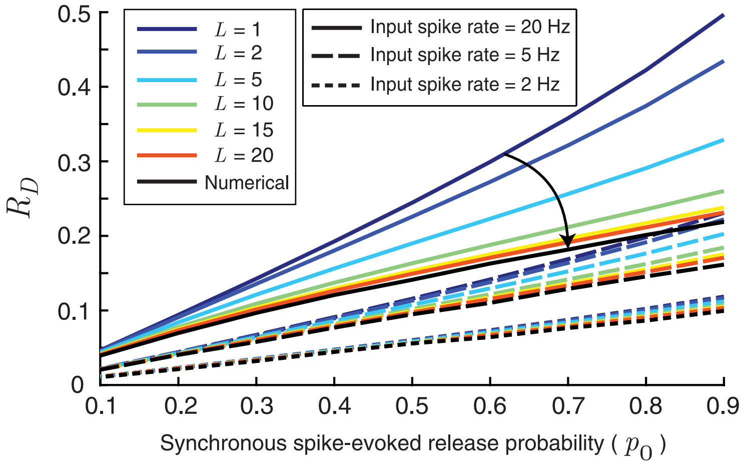

Figure S2: Comparison between the analytical mutual information rate of the MRO model and the numerical estimation of the information rate of a classical stochastic model of depression [1]. The mutual information rate is plotted as a function of synchronous spike-evoked release probability (p_0) for various values of memory length, L, and input spike rates. The recovery time constant of the stochastic model is 100 msec and the corresponding recovery coefficient of the MRO model is $e = 0.1$. In this synapse model, the asynchronous release probability is zero, and the release site is inactivated after each release (i.e., $c = 0$).

Applying the chain rule to $H(Y^n|X^n)$,

$$H(Y^n|X^n) = \sum_{i=1}^{n} H(Y_i|X^n, Y^{i-1}). \tag{23}$$

With a similar argument,

$$H(Y_i|X^n, Y^{i-1}) = H(Y_i|X_i, Y_{i-L}^{i-1}). \tag{24}$$

From (19), (22) and (24),

$$I(X^n; Y^n) = \sum_{i=1}^{n} \big(H(Y_i|Y_{i-L}^{i-1}) - H(Y_i|X_i, Y_{i-L}^{i-1})\big), \tag{25}$$

and based on the definition of conditional mutual information,

$$I(X^n; Y^n) = \sum_{i=1}^{n} I(X_i; Y_i|Y_{i-L}^{i-1}). \tag{26}$$

The sample set of Y_{i-L}^{i-1} consists of all the binary vectors of length L. For the sake of notational simplicity, instead of the binary vector $(Y_{i-L}, ..., Y_{i-2}, Y_{i-1})$, we use its corresponding decimal value $j = Y_{i-1} + 2Y_{i-2} + 2^2 Y_{i-3} + ... + 2^{L-1} Y_{i-L}$. From (26),

$$I(X^n; Y^n) = \sum_{i=1}^{n} \sum_{j=0}^{2^L-1} I(X_i; Y_i|Y_{i-L}^{i-1} = j) P(Y_{i-L}^{i-1} = j). \tag{27}$$

Let R_j represent the mutual information rate of the release site at state j. By definition,

$$R_j = I(X_i; Y_i|Y_{i-L}^{i-1} = j). \tag{28}$$

The release probabilities of the release site at state j, denoted by $p(j)$ and $q(j)$, are calculated from the algorithm in Fig. 1B. It can be easily shown that

$$R_j = h\big(\overline{\alpha} q(j) + \alpha p(j)\big) - \overline{\alpha} h\big(q(j)\big) - \alpha h\big(p(j)\big). \tag{29}$$

Each state of the release site can transit to two other states and the transition probabilities are fully determined by the current state (Fig. 1D). Therefore, a Markov chain is used to model the state transitions of the release site. The transition matrix of the Markov chain, denoted by M, is a $2^L \times 2^L$ matrix and has two non-zero entries on each row. The pattern of the non-zero entries of M is shown in Fig. S3A. It is shown in [4] that for an irreducible aperiodic finite-state Markov chain, regardless of the initial state, the probability of each state j will converge to a steady-state probability, denoted by π_j. We prove in Lemma 1 that the Markov chain of the release site in the MRO model is irreducible and aperiodic. Therefore,

$$\lim_{i\to\infty} P(Y_{i-L}^{i-1} = j) = \pi_j. \tag{30}$$

By interchanging the summations in (27),

$$I(X^n; Y^n) = \sum_{j=0}^{2^L-1} \sum_{i=1}^{n} I(X_i; Y_i | Y_{i-L}^{i-1} = j) P(Y_{i-L}^{i-1} = j). \tag{31}$$

Since $I(X_i; Y_i | Y_{i-L}^{i-1} = j) = R_j$ and is independent of i,

$$I(X^n; Y^n) = \sum_{j=0}^{2^L-1} \left(\sum_{i=1}^{n} P(Y_{i-L}^{i-1} = j) \right) R_j. \tag{32}$$

We then have

$$R_D = \lim_{n\to\infty} \frac{1}{n} I(X^n; Y^n) \tag{33}$$

$$= \sum_{j=0}^{2^L-1} \left(\lim_{n\to\infty} \frac{1}{n} \sum_{i=1}^{n} P(Y_{i-L}^{i-1} = j) \right) R_j. \tag{34}$$

Using (30) and the Cesàro mean theorem,

$$\lim_{n\to\infty} \frac{1}{n} \sum_{i=1}^{n} P(Y_{i-L}^{i-1} = j) = \pi_j. \tag{35}$$

Finally, from (34) and (35),

$$R_D = \sum_{j=0}^{2^L-1} R_j \pi_j. \tag{36}$$

□

We note that the stationary probability vector $\overrightarrow{\pi} = (\pi_0, ..., \pi_{2^L-1})$ is calculated using the power method. We start by a random probability vector $\overrightarrow{x_0}$ and in each iteration $i \geq 0$, we calculate

$$\overrightarrow{x_{i+1}} = \overrightarrow{x_i} \times M. \tag{37}$$

Then we substitute $\overrightarrow{x_i}$ with $\overrightarrow{x_{i+1}}$ and repeat (37). It is easily shown that the probability vector $\overrightarrow{x_i}$ converges to $\overrightarrow{\pi}$.

Lemma 1. *In the MRO model, the markov chain of the release site is irreducible and aperiodic.*

Proof. Let j and j' be two arbitrary states of the release site corresponding to the binary vectors $(a_1, a_2, ..., a_L)$ and $(b_1, b_2, ..., b_L)$. We show that the state j' is always accessible from the state j in the Markov chain M.

Assume that the Markov chain is in the state j at time $i = 1$. The release site can transit to the state $(a_2, a_3, ..., a_L, b_1)$ with a non-zero probability $P_1(b_1)$. Similarly, at each time $i, 1 \leq i \leq L$, the release site can transit from the state $(a_i, ..., a_L, b_1, ..., b_{i-1})$ to $(a_{i+1}, ..., a_L, b_1, ..., b_i)$ with the non-zero probability $P_i(b_i)$. Therefore, the probability of transition from $(a_1, a_2, ..., a_L)$ to $(b_1, b_2, ..., b_L)$ after L time steps is greater than or equal to $\prod_{i=1}^{L} P_i(b_i)$. This proves that the state j' is accessible from the state j, and consequently, M is irreducible. Moreover, there is a non-zero transition probability from the state $j = 0$ to itself. Since every irreducible finite-state Markov chain with a self-loop is aperiodic [4], we conclude that M is aperiodic and the proof is complete.

□

Proof of Theorem 2: The energy-normalized information rate of the release site is defined by (refer to Section A):

$$R_D^{(E)} = \lim_{n\to\infty} \frac{I(X^n; Y^n)}{\mathbf{E}(\sum_{i=1}^{n} E_i)}. \tag{38}$$

where E_i is the energy consumed by the release site to release a vesicle at time i. By assumption, one unit of energy is consumed at each release. Therefore,

$$E_i = Y_i, \tag{39}$$

and

$$\mathbf{E}(\sum_{i=1}^{n} E_i) = \sum_{i=1}^{n} P(Y_i = 1). \tag{40}$$

From (38), (40) and Theorem 1,

$$R_D^{(E)} = \frac{\lim_{n\to\infty} \frac{1}{n} I(X^n; Y^n)}{\lim_{n\to\infty} \frac{1}{n} \sum_{i=1}^{n} P(Y_i = 1)}, \tag{41}$$

$$= \frac{\sum_{j=0}^{2^L-1} R_j \pi_j}{\lim_{n\to\infty} \frac{1}{n} \sum_{i=1}^{n} P(Y_i = 1)}. \tag{42}$$

Also,

$$P(Y_i = 1) = \sum_{j=0}^{2^{(L-1)}-1} P(Y_i = 1, Y_{i-L+1}^{i-1} = j), \tag{43}$$

$$= \sum_{j=0}^{2^{(L-1)}-1} P(Y_{i-L+1}^{i} = 2j + 1). \tag{44}$$

Therefore,

$$\lim_{n\to\infty} P(Y_i = 1) = \sum_{j=0}^{2^{(L-1)}-1} \pi_{2j+1}. \tag{45}$$

From (42), (45) and Cesàro mean theorem,

$$R_D^{(E)} = \frac{\sum_{j=0}^{2^L-1} R_j \pi_j}{\sum_{j=0}^{2^{(L-1)}-1} \pi_{2j+1}}. \tag{46}$$

□

E. Quantized release probabilities

In the MRO model, the release probabilities of the release site at time i are determined by the last L release outcomes, Y_{i-L}^{i-1}. Alternatively, the release probabilities at time i can be derived recursively from the release probabilities and the release outcome at time $i-1$. In this recursive approach, the state of the release site at time i is specified by the pair (P_i, Q_i), where P_i and Q_i are the random variables corresponding to the synchronous spike-evoked and asynchronous release probabilities. Since P_i and Q_i are continuous variables, the number of states goes to infinity by increasing i. To avoid the limitations of infinite-state models, we quantize the release probabilities. For a quantization level of δ, the sample space of release probabilities is defined by

$$S = \{0, \delta, 2\delta, ..., [\frac{1}{\delta}]\delta\}, \tag{47}$$

where [.] is the floor function.

Let $[x]_S$ represent the largest entry in S that is less than or equal to x, i.e.,

$$[x]_S = \max\{y : y \in S, y \leq x\}. \tag{48}$$

Also assume that $p_0, q_0 \in S$ are the default (maximum) synchronous spike-evoked and asynchronous release probability of the release site. The quantized release probabilities at time $i+1$ are calculated recursively from

$$p_{i+1} = \begin{cases} [e(p_0 - p_i) + p_i]_S, & \text{if } Y_i = 0 \\ [cp_i]_S, & \text{if } Y_i = 1 \end{cases} \tag{49}$$

and

$$q_{i+1} = \begin{cases} [f(q_0 - q_i) + q_i]_S, & \text{if } Y_i = 0 \\ [dq_i]_S, & \text{if } Y_i = 1 \end{cases} \tag{50}$$

We refer to this model as the binary asymmetric channel with Quantized Release Probabilities, abbreviated by QRP.

We note that since synchronous spike-evoked and asynchronous release probabilities do not exceed p_0 and q_0, the sample space of (P, Q) can be reduced from $S \times S$ to $S_P \times S_Q$, where

$$S_P = \{0, \delta, 2\delta, ..., p_0\}, \tag{51}$$

and

$$S_Q = \{0, \delta, 2\delta, ..., q_0\}. \tag{52}$$

In the QRP model, the state of the release site at time i, (p_i, q_i), can transit to two other states, the recovered state $([e(p_0-p_i)+p_i]_S, [f(q_0-q_i)+q_i]_S)$ and the depressed state $([cp_i]_S, [dq_i]_S)$, with the transition probabilities $\alpha\overline{p_i} + \overline{\alpha}\,\overline{q_i}$ and $\alpha p_i + \overline{\alpha} q_i$ respectively. The state transitions of the QRP model are represented by a Markov chain with the transition matrix $\widehat{M}$. Unlike the MRO model, the pattern of the non-zero entries of $\widehat{M}$ is not fixed and varies with the model parameters (Fig. S3B). For each state (p, q), the stationary probability, $\pi_{(p,q)}$, is calculated using the power method. Also, the mutual information rate of the binary asymmetric channel, $R_{(p,q)}$, is derived from

$$R_{(p,q)} = h(\overline{\alpha}q + \alpha p) - \overline{\alpha}h(q) - \alpha h(p). \tag{53}$$

Theorem 1. *Let R_D and $R_D^{(E)}$ be the mutual information rate and energy-normalized information rate of the release site in the QRP model. Then*

$$R_D = \sum_{(p,q)\in P_S \times Q_S} R_{(p,q)}\pi_{(p,q)}, \tag{54}$$

$$R_D^{(E)} = \frac{\sum_{(p,q)\in P_S \times Q_S} R_{(p,q)}\pi_{(p,q)}}{\sum_{(p,q)\in P_S \times Q_S}(\alpha p + \overline{\alpha}q)\pi_{(p,q)}}. \tag{55}$$

Proof. Let $X = \{X_i\}_{i=1}^{\infty}$ and $Y = \{Y_i\}_{i=1}^{\infty}$ be the input and output random processes of the QRP model (the top panel in Fig. S3B). By definition,

$$R_D = \lim_{n\to\infty} \frac{1}{n} I(X^n; Y^n), \tag{56}$$

where

$$I(X^n; Y^n) = H(Y^n) - H(Y^n|X^n). \tag{57}$$

Using the chain rule for $H(Y^n)$ and $H(Y^n|X^n)$,

$$H(Y^n) = \sum_{i=1}^{n} H(Y_i|Y^{i-1}), \tag{58}$$

$$H(Y^n|X^n) = \sum_{i=1}^{n} H(Y_i|Y^{i-1}, X^n). \tag{59}$$

The vector of release probabilities, (P_i, Q_i), can be calculated from Y^{i-1}. Also, given X_i and (P_i, Q_i), Y_i is independent of Y^{i-1}, X^{i-1} and X_{i+1}^n. Therefore,

$$H(Y_i|Y^{i-1}, X^n) = H\big(Y_i|(P_i, Q_i), X_i\big). \tag{60}$$

Similarly, given (P_i, Q_i), Y_i is independent of Y^{i-1}, implying that

$$H(Y_i|Y^{i-1}) = H\big(Y_i|(P_i, Q_i)\big). \tag{61}$$

Hence,

$$I(X^n; Y^n) = \sum_{i=1}^{n} H\big(Y_i|(P_i, Q_i)\big) - H\big(Y_i|(P_i, Q_i), X_i\big) \tag{62}$$

$$= \sum_{i=1}^{n} I\big(X_i; Y_i|(P_i, Q_i)\big). \tag{63}$$

From the definition of conditional mutual information,

$$I\big(X_i; Y_i|(P_i, Q_i)\big) = \sum_{(p,q)\in P_S\times Q_S} I\big(X_i; Y_i|(P_i, Q_i) = (p, q)\big) P\big((P_i, Q_i) = (p, q)\big). \tag{64}$$

The term $I\big(X_i; Y_i|(P_i, Q_i) = (p, q)\big)$ is the mutual information rate of the binary asymmetric channel with release probabilities p and q, which is denoted by $R_{(p,q)}$. Therefore,

$$R_{(p,q)} = I\big(X_i; Y_i|(P_i, Q_i) = (p, q)\big) \tag{65}$$

$$= h(\overline{\alpha} q + \alpha p) - \overline{\alpha} h(q) - \alpha h(p). \tag{66}$$

Together with (56), (63) and (64),

$$R_D = \lim_{n\to\infty} \frac{1}{n} \sum_{i=1}^{n} \sum_{(p,q)\in P_S\times Q_S} R_{(p,q)} P\big((P_i, Q_i) = (p, q)\big). \tag{67}$$

By interchanging the summations and moving the limit inside,

$$R_D = \sum_{(p,q)\in P_S\times Q_S} R_{(p,q)} \Big(\lim_{n\to\infty} \frac{1}{n} \sum_{i=1}^{n} P\big((P_i, Q_i) = (p, q)\big)\Big). \tag{68}$$

The state of the release site at time i is given by (P_i, Q_i) and the state transitions of the release site are modeled by a Markov chain with a transition matrix $\widehat{M}$ of order $|P_S| \times |Q_S|$. We prove in Lemma 2 that the Markov chain $\widehat{M}$ is uni-chain and its recurrent class is aperiodic. Therefore, it has a unique stationary distribution and the probability of each state (p, q) converges to its stationary probability $\pi_{(p,q)}$ [4]. That is

$$\lim_{i\to\infty} P\big((P_i, Q_i) = (p, q)\big) = \pi_{(p,q)}. \tag{69}$$

Applying the Cesàro mean theorem to (68),

$$R_D = \sum_{(p,q)\in P_S\times Q_S} R_{(p,q)}\pi_{(p,q)}. \tag{70}$$

Finally, similar to the proof of Theorem 2,

$$R_D^{(E)} = \frac{\sum_{(p,q)\in P_S\times Q_S} R_{(p,q)}\pi_{(p,q)}}{\lim_{n\to\infty}\frac{1}{n}\sum_{i=1}^{n} P(Y_i = 1)}. \tag{71}$$

In the QRP model,

$$P(Y_i = 1) = \sum_{(p,q)\in P_S\times Q_S} P\big(Y_i = 1|(P_i, Q_i) = (p, q)\big)P\big((P_i, Q_i) = (p, q)\big). \tag{72}$$

Hence,

$$\lim_{i\to\infty} P(Y_i = 1) = \sum_{(p,q)\in P_S\times Q_S} (\alpha p + \overline{\alpha} q)\pi_{(p,q)}. \tag{73}$$

From (71), (73) and the Cesàro mean theorem,

$$R_D^{(E)} = \frac{\sum_{(p,q)\in P_S\times Q_S} R_{(p,q)}\pi_{(p,q)}}{\sum_{(p,q)\in P_S\times Q_S}(\alpha p + \overline{\alpha} q)\pi_{(p,q)}}. \tag{74}$$

□

Lemma 2. *The Markov chain of the QRP model, $\widehat{M}$, is uni-chain and its recurrent class is aperiodic.*

Proof. To show that the transition matrix of the QRP model is uni-chain, we need to prove that there exists only one recurrent class in $\widehat{M}$ and the other states (if any) are transient.

Let $(a_0, b_0) \neq (0, 0)$ be an arbitrary state in $\widehat{M}$. Consider the path $(a_0, b_0) \to (a_1, b_1) \to (a_2, b_2) \to ...$ in which every state (a_i, b_i) transits to its depressed state, i.e., $(a_{i+1}, b_{i+1}) = ([ca_i]_S, [db_i]_S)$. As long as $(a_i, b_i) \neq (0, 0)$, the transition probability to the depressed state, (a_{i+1}, b_{i+1}), is positive. Moreover, if $a_i > 0$ then $a_{i+1} < a_i$, and if $b_i > 0$ then $b_{i+1} < b_i$. Since the number of states in the Markov chain is finite and the sequences $(a_i)_{i\in\mathbb{N}}$ and $(b_i)_{i\in\mathbb{N}}$ are monotonically decreasing to zero, there will be a large enough integer N such that $(a_N, b_N) = (0, 0)$. Therefore, there is a path from (a_0, b_0) to the state $(0, 0)$ in $\widehat{M}$. This implies that the state $(0, 0)$ is accessible from every state in the Markov chain.

Now assume that there are two recurrent classes C_1 and C_2 in the Markov chain. Since the states in C_1 have access to $(0, 0)$, from the definition of recurrent states, $(0, 0) \in C_1$. With a similar argument, $(0, 0) \in C_2$. Therefore, $C_1 = C_2$ and there is only one recurrent class in $\widehat{M}$, meaning that $\widehat{M}$ is a uni-chain.

Now we show that the period of the recurrent class is equal to one. Since the transition probability to the recovered state is always positive in the Markov chain $\widehat{M}$, we can consider the path $(a_0, b_0) = (0, 0) \to (a_1, b_1) \to (a_2, b_2) \to ...$ in which every state (a_i, b_i) transits to its recovered state, i.e., $(a_{i+1}, b_{i+1}) = ([e(p_0 - a_i) + a_i]_S, [f(q_0 - b_i) + b_i]_S)$. It is clear that for each i, $a_{i+1} \geq a_i$ and $b_{i+1} \geq b_i$. Since the number of states is finite, there exists a finite integer N such that

$$a_N = [e(p_0 - a_N) + a_N]_S, \tag{75}$$

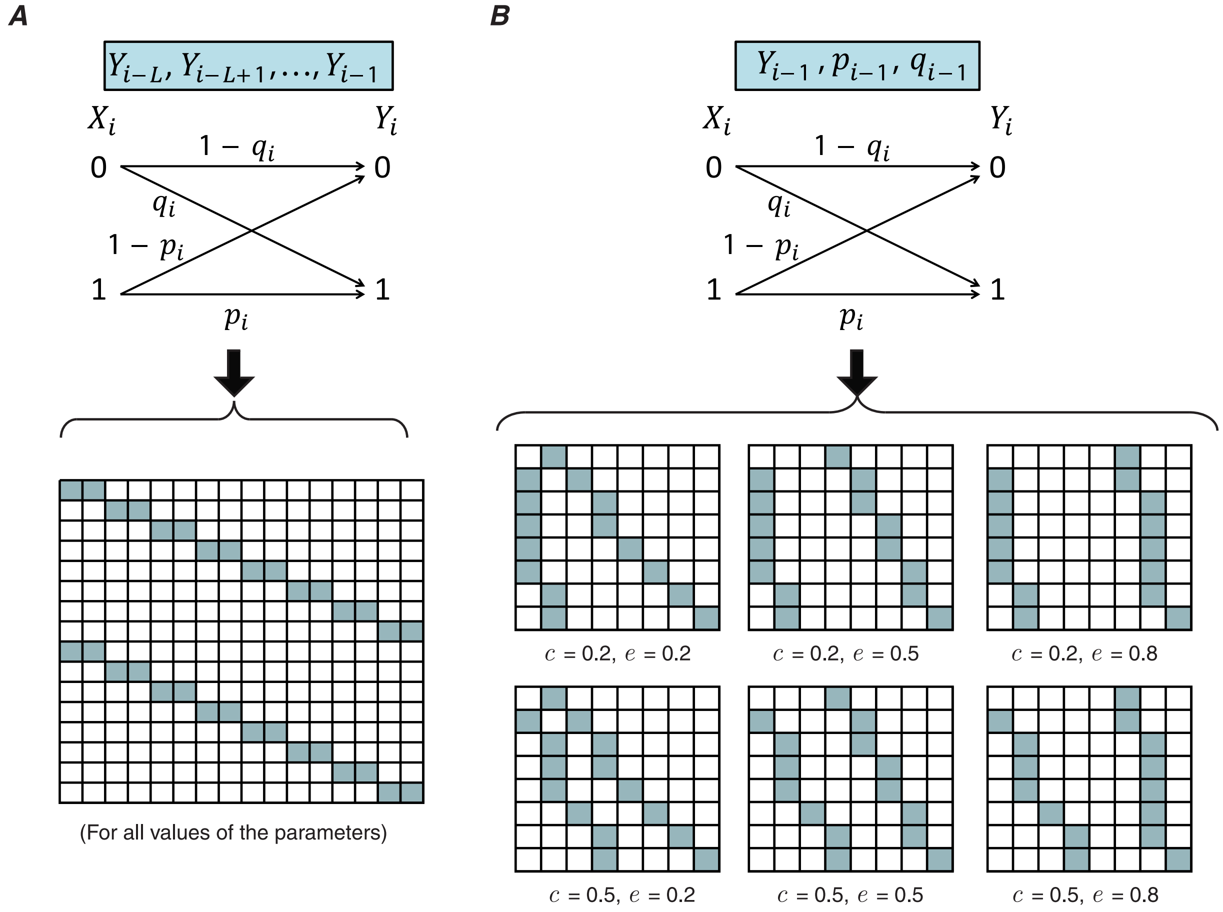

Figure S3: The non-zero entries of the transition matrix in the two models. (A) The MRO model with a memory of length $L = 4$. The transition matrix is invariant and does not change by altering the model parameters. (B) The QRP model. The pattern of the transition matrix varies based on the model parameters. The first row shows the pattern for fixed depression multiplier ($c = 0.2$) and different recovery coefficients. The second row shows the variation of the pattern for $c = 0.5$. The other parameters are $\alpha = 0.6$, $p_0 = 0.7$, $q_0 = 0.1$, $d = c$, and $f = e$.

$$b_N = [f(q_0 - b_N) + b_N]_S. \tag{76}$$

Therefore, the state (a_N, b_N) transits to itself with probability $\alpha\overline{a_N} + \overline{\alpha}\,\overline{b_N}$. On the other hand, (a_N, b_N) is accessible from $(0, 0)$, meaning that it belongs to the recurrent class. Since a recurrent state with a loop is aperiodic [4], we conclude that (a_N, b_N), and consequently the recurrent class of $\widehat{M}$, is aperiodic and the proof is complete. □

F. Comparison between the two models of short-term depression

We presented two models to calculate the mutual information rate of the release site during short-term depression: the MRO model and the QRP model. The mutual information rates and energy-normalized information rates of the two model are similar (compare Fig. S4A to Fig. 3B). We show in Fig. S4B that the relative difference between the calculated rates of the two models is negligible. Each model, however, has its own advantages and disadvantages. The state of the release site in the MRO model is a binary vector of length L, which corresponds to the last L release outcomes. The Markov chain of the release site consists of 2^L states and its transition matrix, M, grows exponentially with memory length. The pattern of non-zero entries in M is always fixed and does not depend on depression dynamics (Fig. S3A). In contrast, the state

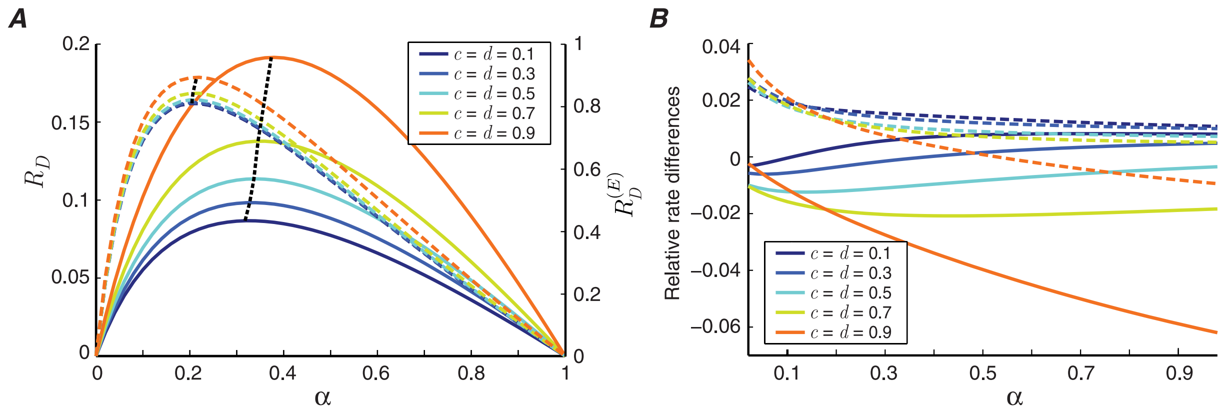

Figure S4: (A) For the QRP model, the mutual information rate (solid lines) and the energy-normalized information rate (dashed lines) are plotted as a function of input spike rate, for various depression multipliers. The other parameters of the model are $e = f = 0.1$, $p_0 = 0.7$ and $q_0 = 0.1$. (B) The relative difference between the information rates of the MRO model and the QRP model, as a function of input spike rate, for various values of depression multiplier. The solid lines show the relative difference of mutual information rates, $\frac{R_D(QRP)-R_D(MRO)}{R_D(MRO)}$, and the dashed lines show the relative difference of energy-normalized information rates, $\frac{R_D^{(E)}(QRP)-R_D^{(E)}(MRO)}{R_D^{(E)}(MRO)}$. The parameters of the two models are similar to (A).

space of the QRP model consists of the quantized release probabilities, which is modeled by a Markov chain, $\widehat{M}$, of order $|S_P|\times|S_Q|$. Therefore, the size of the Markov chain in the QRP model can be much smaller than that of the MRO model. This will decrease the computational resources that are required for calculation of information rate in the QRP model. However, the pattern of non-zero entries of $\widehat{M}$ varies with depression coefficients (Fig. S3B), making it more difficult to achieve further analytical advances.

G. Distinct pools of vesicles

Studies indicate that asynchronous and synchronous release rely on the same pool of the vesicles, whereas spontaneous release may draw on a distinct pool.

Here we consider the hypothetical scenario in which the rate of spontaneous release is similar to that of asynchronous release to explore the consequences of having distinct pools of vesicles. Although our framework is based on the notion of a shared pool of vesicles, it can be slightly modified to comprise these hypothetical cases as well. We calculate average depression multipliers, $\bar{c}$ and $\bar{d}$, by marginalizing c and d over the release modes:

$$\bar{c} = \frac{c\big(\alpha p_a + (1-\alpha) r q_a\big) + (1-\alpha)(1-r)q_a}{\alpha p_a + (1-\alpha)q_a}, \tag{77}$$

$$\bar{d} = \frac{\big(dr + (1-r)\big)\big(\alpha p_a + (1-\alpha) r q_a\big) + \big(r + d(1-r)\big)(1-\alpha)(1-r)q_a}{\alpha p_a + (1-\alpha)q_a}, \tag{78}$$

where p_a is the average synchronous release probability, q_a denotes the average asynchronous plus spontaneous release probability, and r determines the ratio of the number of asynchronous releases to the total number of releases in the inter-spike intervals. The average depression multipliers substitute c and d in the model and simulate the impact of distinct pools of vesicles.

We study the extreme case for which the rates of spontaneous release and asynchronous release are identical, $r = 0.5$. If spontaneous releases come from a distinct pool of vesicles, the release of a vesicle after an action potential depresses both synchronous and asynchronous release, but does not change the spontaneous release probability. For releases occurring in the inter-spike intervals, if the released vesicle belonged to the separate pool of spontaneous release, only the probability of spontaneous release is reduced

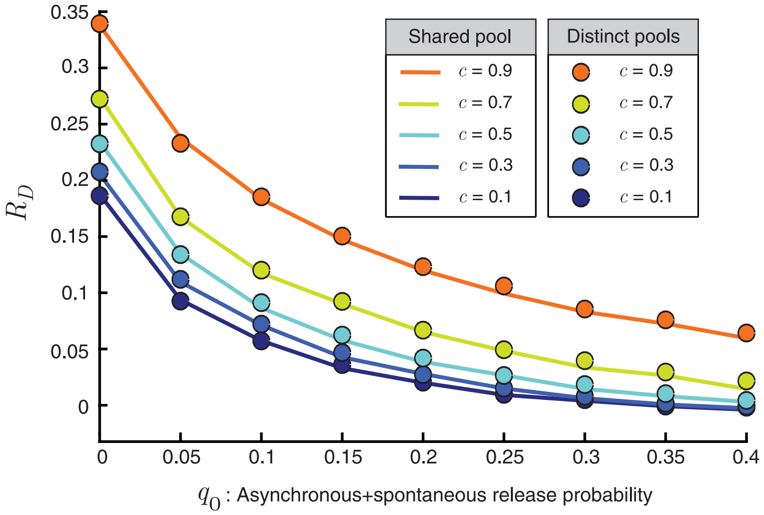

Figure S5: Information efficacy of a synapse with separate pools of vesicles. The mutual information rate of a synapse with a distinct pool of vesicles for spontaneous release is calculated numerically using context tree weighting algorithm (filled circles). The average depression multipliers of the MRO model (with a shared pool of vesicles) are estimated and the mutual information rate of the synapse is calculated (solid lines). Information rates are plotted as a function of q_0 (the summation of asynchronous and spontaneous release probabilities) for different depression multipliers of synchronous release, c. The other parameters are $d = 0.5, a = 0.2, p_0 = 0.7$, and $e = f = 0.1$.

and the other modes of release are not affected; otherwise, depression reduces the probabilities of synchronous and asynchronous release, but does not impact spontaneous release.

The mutual information rate of the synapse is calculated numerically using the context-tree weighting algorithm [2]. We then update the MRO model with average depression multipliers and calculate the mutual information rate of the synapse. As seen in Fig. S5, even in this extreme case (with an unrealistically high rate of spontaneous release), the mutual information rate of the synapse with a distinct vesicle pool for spontaneous release can be precisely estimated by the MRO mode (with a shared pool of vesicles), provided that depression multipliers are marginalized over the release modes.

We should note that the same simulation can be used to study a hypothetical synapse in which half of the vesicles of asynchronous release are supplied from a separate pool of vesicles. Our results show that the MRO model, equipped with average depression multipliers, can provide an accurate estimation of the information efficacy of synapses with partially distinct pools of vesicles.

References

1. Fuhrmann G, Segev I, Markram H, Tsodyks M. Coding of temporal information by activity-dependent synapses. Journal of Neurophysiology. 2002;87(1):140–148.
2. Jiao J, Permuter HH, Zhao L, Kim YH, Weissman T. Universal estimation of directed information. IEEE Transactions on Information Theory. 2013;59(10):6220–6242.
3. Cover TM, Thomas JA. Elements of information theory. John Wiley & Sons; 2012.
4. Gallager RG. Discrete stochastic processes. vol. 321. Springer Science & Business Media; 2012.
